# Supplementary material for: Real-time encoding and compression of neuronal spikes by metal-oxide memristors
Source: Nat Commun. 2016 Sep 26;7:12805. doi: 10.1038/ncomms12805 (PMC5052668; doi:10.1038/ncomms12805)
Supplement: Supplementary Information — Supplementary Figures 1 - 13, Supplementary Tables 1 and 2 and Supplementary References [file ncomms12805-s1.pdf]

SUPPLEMENTARY INFORMATION

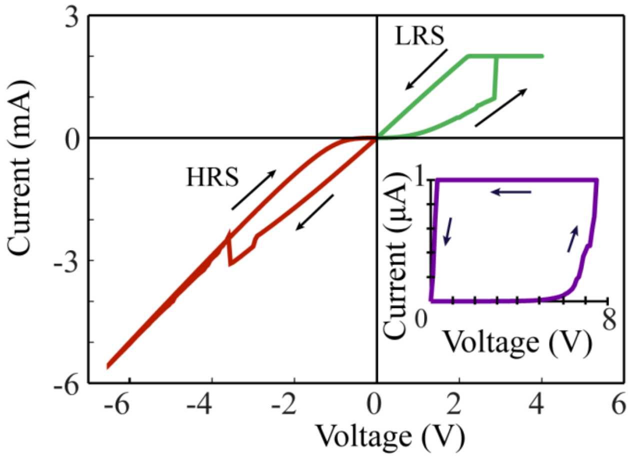

Supplementary Figure 1 DC characterization of the electroformed device, demonstrating a typical pinched-hysteresis memristive device signature. Voltages are applied to the top electrode (TE) whilst the bottom electrode (BE) is grounded for all the measurements. The inset shows the electroforming step where the device forms to 'ON' state under positive bias, shown in purple colour. After electroforming, the device exhibits bipolar switching with low resistive state (LRS, illustrated in green) to high resistive state (HRS, illustrated in red) transitions at ±3V. RESET occurs under negative bias whilst the complementary SET transitions occur under positive bias.

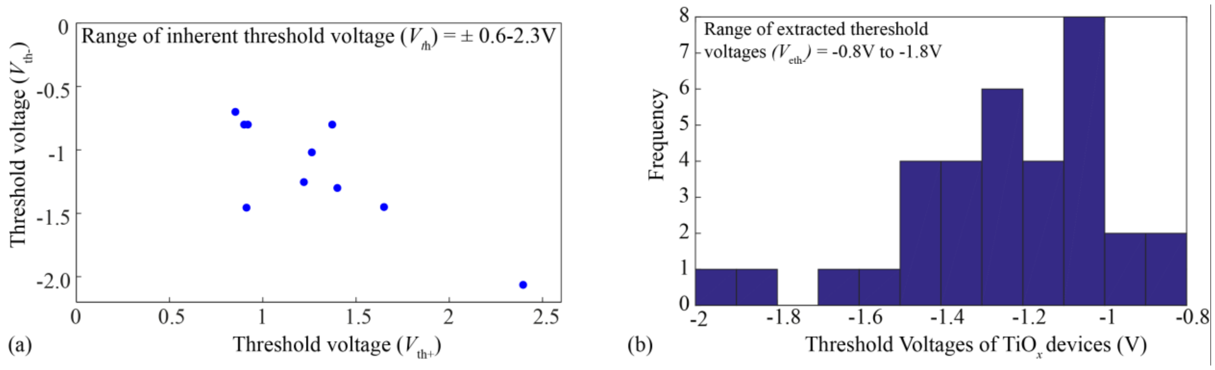

Supplementary Figure 2 Inherent and effective threshold of the memristive devices. (a) Range of the 'inherent' threshold voltage (V_{th-}/V_{th+}) is approximately ± 0.6-2.3V as determined by the sample of data obtained from ten devices. (b) Distribution of effective threshold voltage of the TiO_x devices used. This value is used as an approximation for the setting of gain (G) and offset (V_{off}) parameters for the processing of neural recordings before it is used for biasing of the memristive device. The histogram is plotted for a sample of 34 devices. Range of the effective threshold voltages used for the experiment is -0.8V to -1.8V. The mean (µ) of the sampled data is equal to -1.4V. The one sigma standard deviation values are equal to (-1V, -1.8V).

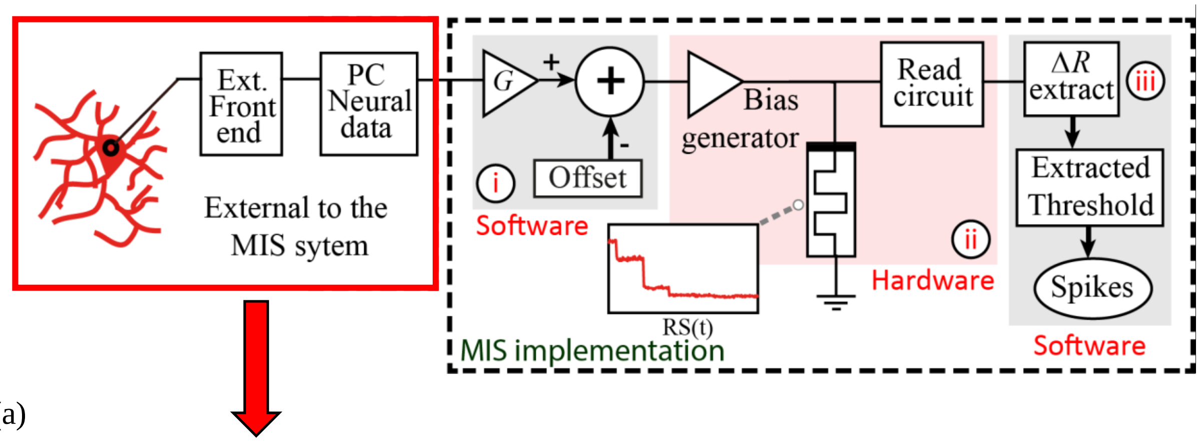

(a)

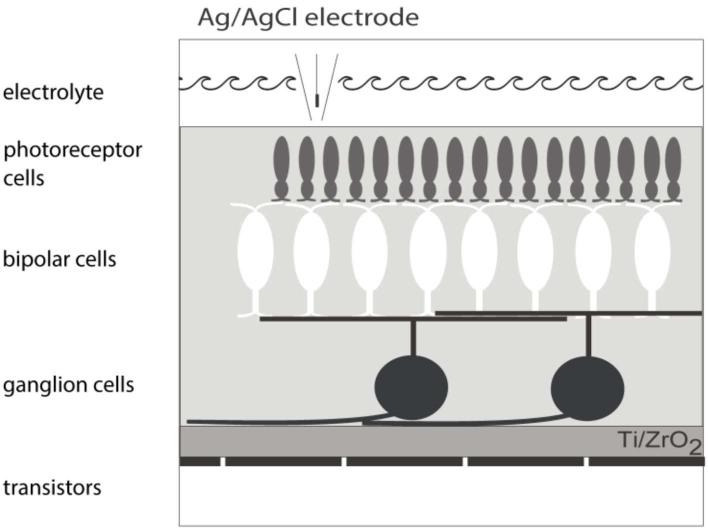

(b)

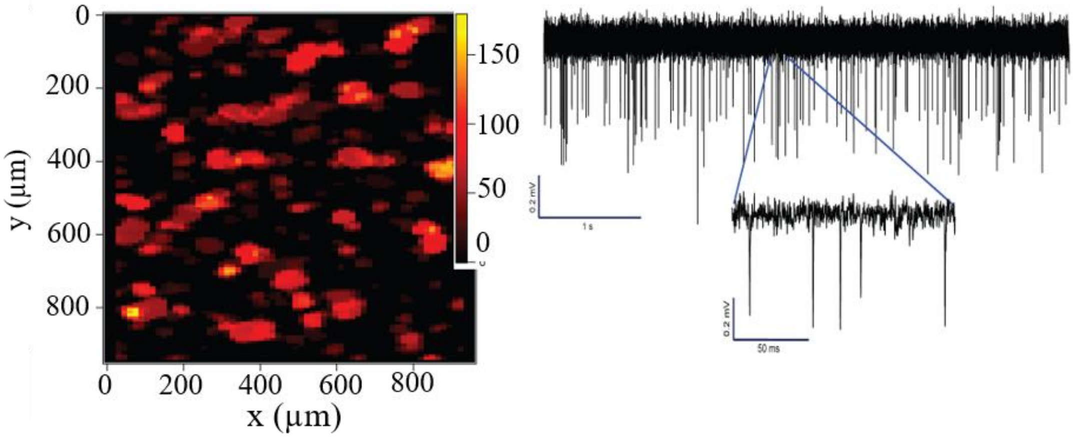

(c)

Supplementary Figure 3 (a) An overall block-diagram of the system used in the neural signal processing through memristive integrating sensors. The front-end system presented in a red rectangle is used and is external to the MIS platform. The black-dashed lines indicate the schematic for the implementation of MIS platform as described in the main manuscript. (b) Illustration of the retina/chip configuration depicting the multi-electrode arrays upon which tissue slices from retinal ganglion cells are placed and measured directly. (c) (Left) Colour coded activity map of electrical activity from tissue slices of rabbit ganglion cells placed atop the MEA. (Right) Traces of pre-amplified data (voltage-time series) obtained from the front-

end set-up in the range of 100's of mV. These recordings are acquired and pre-processed using software-defined (i) gain and offset stage, (ii) the suitably amplified neural recording is then used to bias the memristive devices using the customised hardware illustrated in Fig. S4 and, (iii) the 'read' resistive state values are post-processed off-line to extract spiking activity.

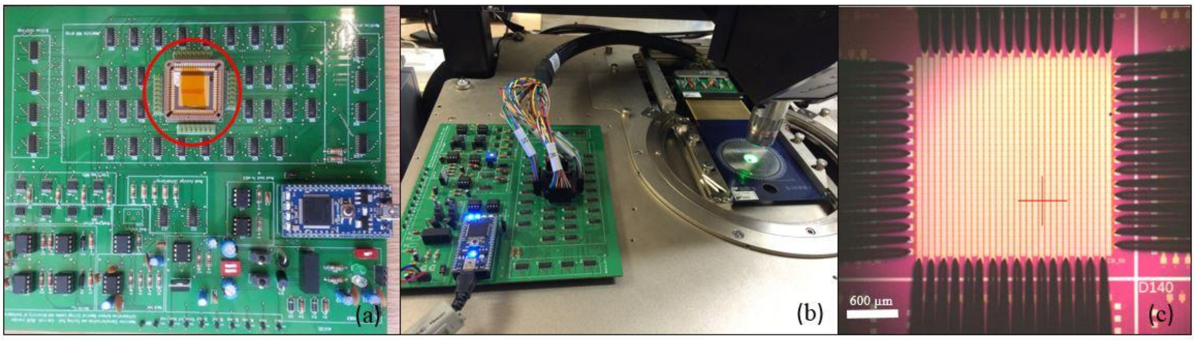

Supplementary Figure 4 Hardware Infrastructure used in order to implement proposed biasing protocol[1,2,3] . (a) PCB-mounted system overview with visible crossbar package (circled in red). (b) System connected to multi-channel probe card. (c) Microscope image of 32x32 crossbar showing probe card needles touchdown.

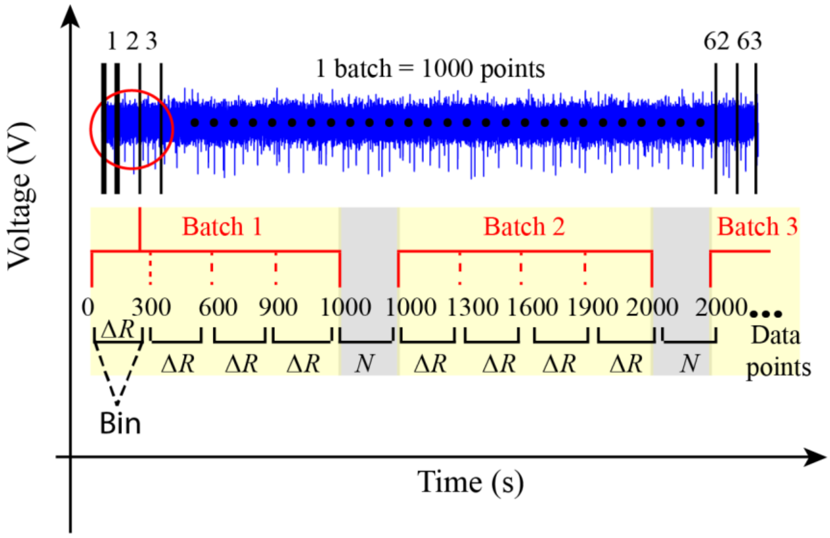

Supplementary Figure 5 Standard read-out scheme for evaluating the time evolution of the resistive state of test devices subjected to input stimulation. A single neural recording contains exactly 63016 data-points. Input data is fed to each device in batches of 1000 points with RS assessed at the beginning of each batch, then every 300 samples and finally at the end of each batch. A single neural recording consists of 63 batches. Changes in resistive state (ΔR) are extracted from consecutive resistive state assessments. Resistive state changes occurring between the last measurement of each batch and the first measurement of the next batch, i.e. with no interceding pulse biasing, (N) are considered to result from measurement uncertainty and can be used to determine the 'noise band'. Thus, we obtain 316 ΔR values, corresponding to 252 ΔR bins and 64 noise samples which helps in determining the effective thresholds separating significant from insignificant RS switching activity ('the noise band').

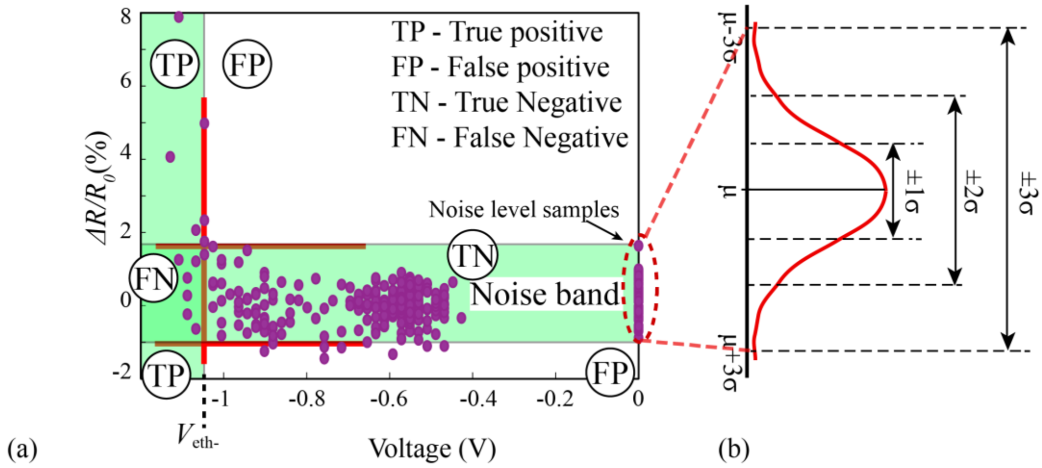

Supplementary Figure 6 Estimation of effective threshold voltage (V_{eth-}) and quantification of data obtained from the MIS platform. (a) Normalised plot of resistive state changes (ΔR) values in each bin is plotted as a function of maximum voltage magnitude of interceding events. (b) Illustration of 6 sigma method used for the setting of the 'noise band' limits. Noise level samples are measured at the end of each batch and the beginning of next batch with no interceding biasing samples. The noise band consists of noise samples and insignificant RS changes arising as a result of weak amplitude neural signals which cannot be differentiated from the insignificant resistive state changes. Everything outside the noise band is estimated as a significant change in the resistive state and is recorded in the output of the MIS system as a detected spike. Notably, the estimation of V_{eth-} is determined by the noise band limits which classifies the data neatly in four groups as illustrated. In a simple thresholded integrator, everything above the threshold is assumed to be a spike and everything below is suppressed as noise. However, due to existence of noise band in the MIS platform, the data in MIS platform is quantified as follows: Outside the noise band and below V_{eth-}: True Positives (TP), outside the noise band and above V_{eth-}: False Positives (FP), inside the noise band and below V_{eth-}: False Negatives (FN), inside the noise band and above V_{eth-}: True Negatives. The results are benchmarked against widely used state-of-the-art template matching system. We assume the template-matching system to be a perfect spike detector in this case.

$$\text{Rate of TP} = \frac{TP}{TP+FP}$$

$$\text{Rate of FP} = \frac{FP}{FP+TN}$$

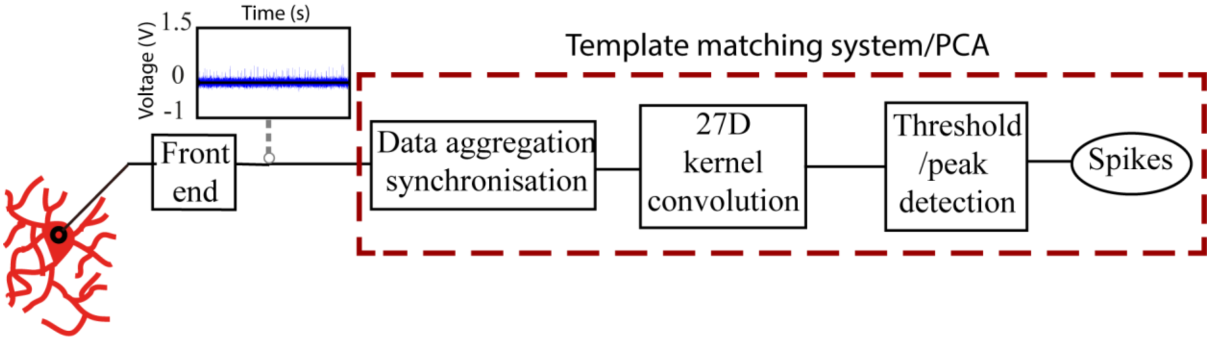

Supplementary Figure 7 Template matching system used as a benchmarking standard for the MIS platform. The front-end system for recording neural data from retinal ganglion cells remains the same for both the systems.

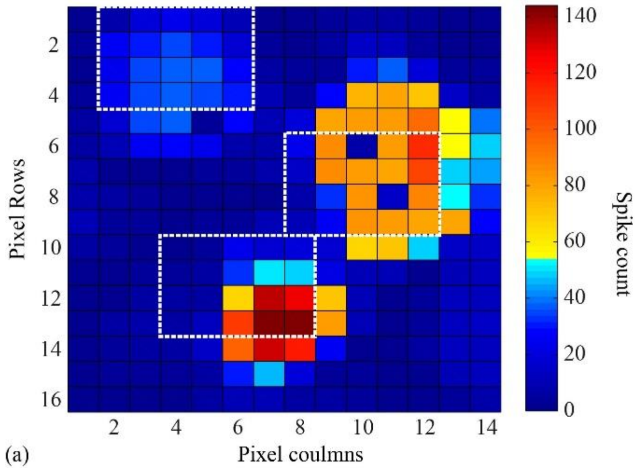

Supplementary Figure 8 Spike count pixel map yielded by the conventional system[4,5] for the MEA sub-array recordings used to obtain Fig. 3c in the main text. Following a conventional signal processing paradigm, template-matching system utilises standard principle component analysis method to identify firing cells[6]. Three major regions of activity can be clearly seen, which roughly correspond to the three main regions of activity detected by the memristive system (marked by white, dashed lines) in Fig. 5d. Colour bar mapping has been chosen to correspond to the one employed in main text Fig. 5.

Row ID / Column ID

S1_Cx_Ry	C30	C32	C34	C36	C38	C40	C42	C44	C46	C48	C50	C52	C54	C56
R82	1	17	33	49	65	81	97	113	129	145	161	177	193	209
R83	2	18	34	50	66	82	98	114	130	146	162	178	194	210
R84	3	19	35	51	67	83	99	115	131	147	163	179	195	211
R85	4	20	36	52	68	84	100	116	132	148	164	180	196	212
R86	5	21	37	53	69	85	101	117	133	149	165	181	197	213
R87	6	22	38	54	70	86	102	118	134	150	166	182	198	214
R88	7	23	39	55	71	87	103	119	135	151	167	183	199	215
R89	8	24	40	56	72	88	104	120	136	152	168	184	200	216
R90	9	25	41	57	73	89	105	121	137	153	169	185	201	217
R91	10	26	42	58	74	90	106	122	138	154	170	186	202	218
R92	11	27	43	59	75	91	107	123	139	155	171	187	203	219
R93	12	28	44	60	76	92	108	124	140	156	172	188	204	220
R94	13	29	45	61	77	93	109	125	141	157	173	189	205	221
R95	14	30	46	62	78	94	110	126	142	158	174	190	206	222
R96	15	31	47	63	79	95	111	127	143	159	175	191	207	223
R97	16	32	48	64	80	96	112	128	144	160	176	192	208	224

Supplementary Figure 9 Overview of the MEA 16x14 sub-array recordings. Green cells: MEA column coordinates of recordings employed. Orange cells: MEA row coordinates of

recordings employed. Blue cells: unique pixel identifier numbers. Dark blue cells indicate recordings that contain at least 1 data sample with magnitude above 0.5V; this includes a number of recordings that barely pass the 0.5V mark. The map therefore represents the presence of spikes as benchmarked by simple threshold method. The 23 recordings shown in orange colour were chosen as a representative sample of the entire array and were then used to estimate gain (G) and offset values (V_{off}) for the main experiments.

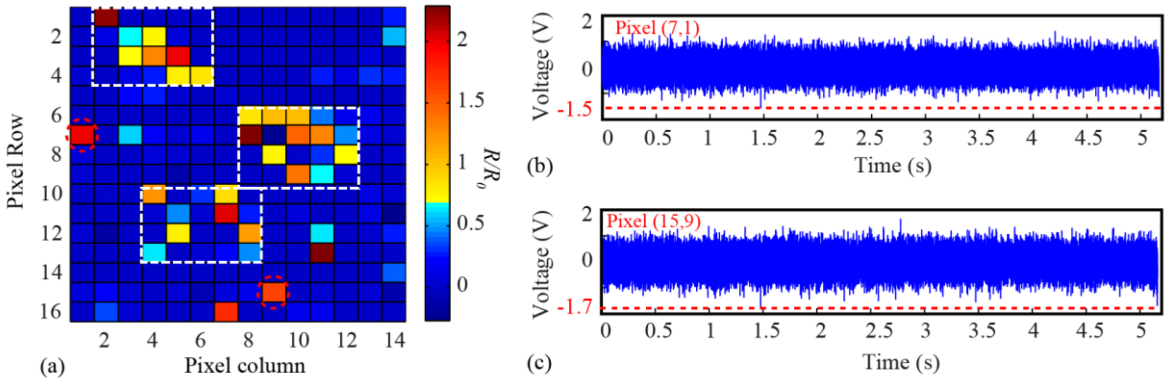

Supplementary Figure 10 The origin of 'stray' pixels in the final output state of memristive integrating sensor as shown in Fig. 5d in main text. (a) Memristive device array final state pixel map reproduced from main text Fig. 5d. It shows the DUT final/initial resistive state ratio. The three main regions of activity are enclosed in white, dashed line boxes, as are two of the four 'stray' pixels that show remarkable change in state without belonging to any of the three main clusters. (b) and (c) We investigated the neural recordings corresponding to these stray pixels and found that they all contain lone, remarkably high amplitude events i.e. -1.5V for the former and -1.7V for the latter; examples from two such recordings are circled in red. The stray pixels therefore react to real events in the stimulus waveform.

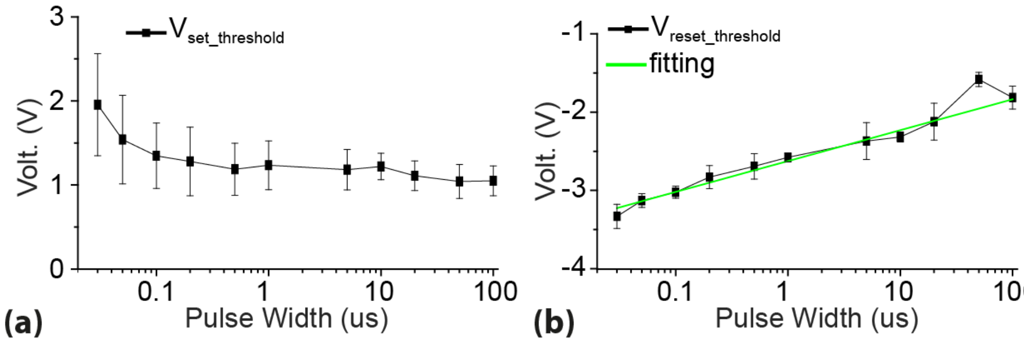

Supplementary Figure 11 Voltage-time trade-off in TiO_x memristors. Estimated threshold voltages for SET RS transitions (transitions towards lower resistive state[7] - a) and for RESET transitions (b) are shown as a function of applied pulse durations. In the case of RESET transitions, we see a good exponential fit, whist in the case of SET transitions the relation grows faster-than-exponentially. In both SET and RESET cases we notice that threshold voltage magnitudes increase only modestly as pulse duration is drastically reduced. Standard error bars

shown. Threshold voltages are computed by applying a series of progressively higher amplitude voltage pulses to the DUT (fixed duration 100 μs) and monitoring resistive state changes. The applied voltage pulse at the moment when DUT RS changes by more than 2% vs. its state at the start of the pulsing session is considered an estimate of voltage threshold.

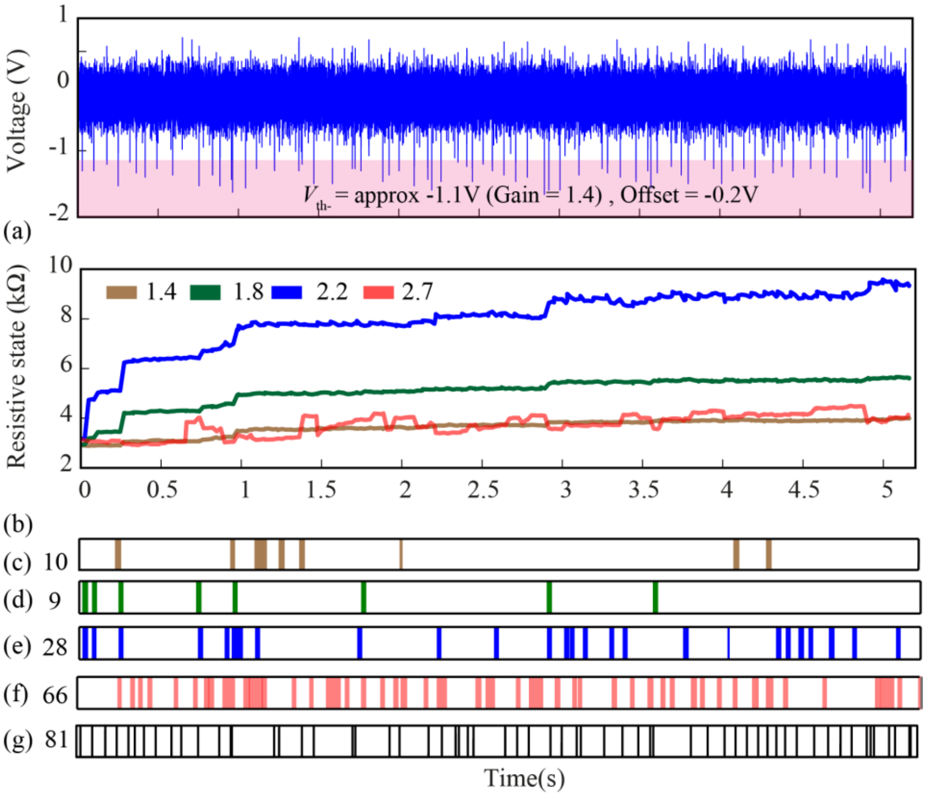

Supplementary Figure 12 Optimisation of gain and offset settings. Comparison of different gain values for one neural recording and the spike count detected by the MIS. (a) Neural recording employed for the test. (b) The time evolution of device-under-test resistive state with gain 1.4, 1.8, 2.2 and 2.7. The offset for the first three gains is -0.2V and +0.4V for the last. (c), (d), (e) and (f) Spike locations and count as detected by MIS system when the gain is set to 1.4, 1.8, 2.2 and 2.7 respectively. (g) Spike locations and count as calculated by the state-of-the-art template matching system.

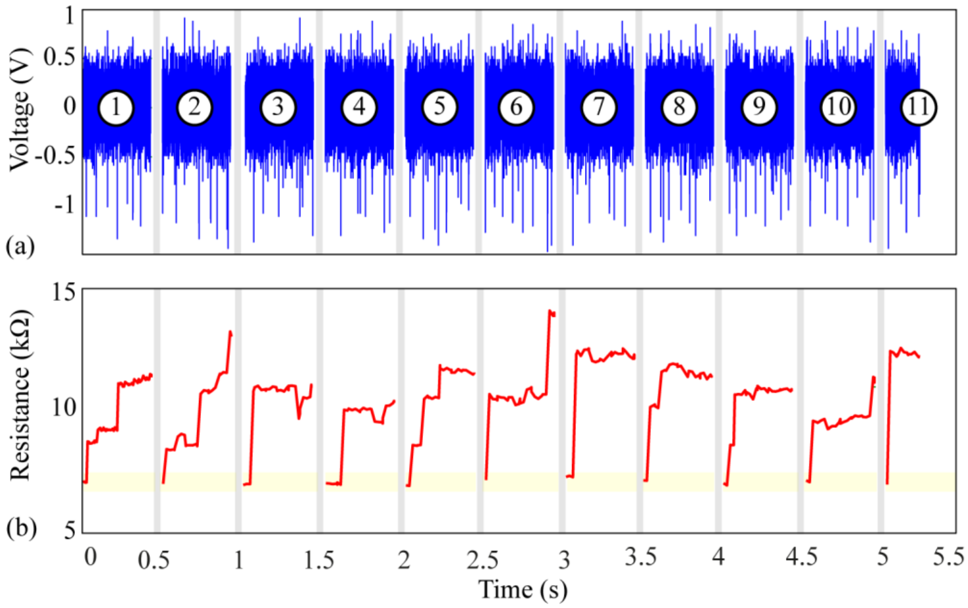

Supplementary Figure 13 Regular resetting of the memristive device to counter the saturation of resistive state of device in order to improve the spike-count of the system. (a) Slicing of neural recording in eleven parts lasting for 0.5s separated by grey bands. The device is manually reset to its initial state after each run (yellow bands). Ron (low resistive state) of the device is approximately equal to 6-8 kΩ. (b) Output of the device when biased with the sub-neural recording in (a) with G = 2.2 and V_{off} = -0.2. Quantification and benchmarking of the above results against the template-matching system indicated a rate of true positives of up to 80% for individual slices.

Fitting coefficients (95% confidence upper/lower bounds)		
Coefficient	Fig.1c	Fig.1d
A	4780 (4756, 4803)	1301 (1233, 1368)
β	$1.878e^{-4}$ ($1.492e^{-4}$, $2.264e^{-4}$)	$-1.027e^{-1}$ ($-1.118e^{-1}$, $-9.366e^{-2}$)
B	-1075 (-1156, -994.7)	2976 (2954,2998)
ϒ	$-1.04e^{-1}$ ($-1.17e^{-1}$, $-9.098e^{-2}$)	$-7.829e^{-4}$ ($-8.418e^{-4}$, $- 7.24e^{-4}$)
Goodness of fit:		
R-square	0.9137	0.9701
Adjusted R-square	0.9124	0.9697
RMSE	56.77	47.8

Supplementary Table 1 The DUT responses shown in Fig.1d and 1e were fitted with second order exponential functions of input voltage integral taking the form $f(\int V dt) = Ae^{\beta \int V dt} + Be^{\Upsilon \int V dt}$, where V is the fixed pulse voltage, using standard MATLAB curve-fitting tool. However under constant voltage pulse stimulation, results yielded by the non-thresholded model can be exactly reproduced by a thresholded model of the form $f(x) = Ae^{\beta'(\int \max(|V-V_{th}|,0)\, dt)} + Be^{\Upsilon'(\int \max|V-V_{th}|,0)\, dt}$ where $\beta' = \beta|V/(V-V_{th})|$, $\Upsilon' = \Upsilon|V/(V-V_{th})|$. This holds for $|V|>|V_{th}|$. Statistical parameters extracted from curve-fitting are summarised in the table above. The fitted curve is in good agreement with the experimental data.

S.No. (from Fig.S6)	TMS	MIS	TP	FP	TN	FN	Rate of TP (%)	Rate of FP (%)
'One device-to two neural recordings' (Fig 4 (a), (b))								
Recording I	20	14	11	3	230	3	52.3	1.28
Recording II	77	52	32	20	156	45	41.5	11.3
'Many devices-one neural recording' (Fig 4.(c), (d))								
Device 1 III	78	40	29	11	164	49	37.2	6.2
Device 2 IV	78	33	26	7	168	52	33.4	4

Supplementary Table 2 Spike count and quantification measures for the neural recordings in Figure 4 in the main text in comparison to the state-of-the-art template matching system (TMS). Spikes detected by Memristive Integrating sensor (MIS) and Template matching system (TMS). True positives: TP, False Positives: FP, True negatives: TN, False negatives: FN.

Supplementary References

1. Gupta, I., Serb, A., Berdan, R, Khiat, A, Regoutz, A & Prodromakis, T. A Cell Classifier for RRAM Process Development. *IEEE Trans. Circuits Syst. II Express Briefs* **62,** 676–680 (2015).

2. Serb, A., Berdan, R., Khiat, A., Papavassiliou, C. & Prodromakis, T. Live demonstration : A versatile , low-cost platform for testing large ReRAM cross-bar arrays . *IEEE Int. Symp. CIRCUITS Syst.* **9,** 4799 (2014).

3. Berdan, R., Serb, A., Khiat, A., Regoutz, A, & Papavassilliou, C. A u-Controller-Based System for Interfacing Selectorless RRAM Crossbar Arrays. *IEEE Trans. Electron Devices* **62,** 2190–2196 (2015).

4. Lichtsteiner, P., Posch, C. & Delbruck, T. A 128x128 120dB 15μs Latency Asynchronous Temporal Contrast Vision Sensor. *IEEE J. Solid-State Circuits* **43,** 566–576 (2008).

5. Eversmann, Bjoern, Hofmann, Franz, Holzapfl, Birgit, Schmitt-landsiedel, D. & Lambacher, Armin, Zeitler, Ralf, Merz, Matthias, Kunze, A. CMOS Sensor Array for Electrical Imaging of Neuronal Activity. *IEEE Int. Symp. CIRCUITS Syst.* **4,** 3479–3482 (2005).

6. Lambacher, A., Vitzthum, V., Zeitler, R. & Eickenscheidt, M., Eversmann, B., Thewes,

R., Fromherz, P. Identifying firing mammalian neurons in networks with high-resolution multi-transistor array (MTA). *Appl. Phys. A* **102,** 1–11 (2010).

7. Xing, J., Serb, A., Berdan, R., Xui, H. & Prodromakis, T. An FPGA-based instrument for en-masse RRAM characterisation with ns pulsing resolution. *IEEE Trans. Circuits Syst. I Regul. Pap.* **63,** 818-826 (2016).
